# Complete Genome Sequence of a South Korean Isolate of *Habenaria mosaic virus*

**DOI:** 10.1128/genomeA.00958-16

**Published:** 2016-09-08

**Authors:** Davaajargal Igori, Seungmo Lim, Fumei Zhao, Dasom Baek, Jae Sun Moon

**Affiliations:** a Biosystems and Bioengineering Program, University of Science and Technology, Daejeon, South Korea; b Molecular Biofarming Research Center, Korea Research Institute of Bioscience and Biotechnology, Daejeon, South Korea

## Abstract

*Habenaria mosaic virus* (HaMV), a member of the genus *Potyvirus* in the family *Potyviridae*, was first discovered from *Habenaria radiata* in Japan*.* The complete genomic sequence of a South Korean isolate (PA1) of HaMV infecting *Plantago asiatica* L. was determined with high-throughput RNA sequencing.

## GENOME ANNOUNCEMENT

*Plantago asiatica* L., a species of the genus *Plantago* in the family *Plantaginaceae*, commonly called “Asian plantain,” is a very familiar weed in areas of East Asia (China, Japan, South Korea, etc.). Traditionally, this plant has been used in Asian pharmacopeia for the treatment of many diseases (1). In July 2011, one *Plantago asiatica* L. plant showing systemic mosaic symptoms was collected in Jeju Island, South Korea. High-throughput RNA sequencing was used to identify the causal agent of the disease. RNA sequencing was performed on an Illumina HiSeq 2500 sequencing system by the Theragen Bio Institute (Suwon, South Korea). The resulting sequences were trimmed and filtered for adapter contamination and low quality. The high-quality filtered next-generation sequencing reads were assembled *de novo* using the Trinity version 2.1.1 program. The assembled contigs were subjected to BLAST search analysis against the GenBank database. Among them, six contigs were identified, ranging from 692 to 1,936 bp, and showing strong similarity to *Habenaria mosaic virus* (HaMV; Ha-1) at the nucleotide level (ranging from 78% to 94% identity) (2). To distinguish the *P. asiatica* L.–infecting virus from the *P. asiatica* L. sample and to confirm and determine the complete genome of the virus, primers were designed based on the six contigs. Total RNA was extracted from the *P. asiatica* L. sample. Complementary DNA (cDNA) was synthesized with a random N25 primer (3), using RevertAid reverse transcriptase (Thermo Scientific, Waltham, MA, USA). The viral cDNA was amplified with PCR using six diagnostic primer pairs. All of the fragments were successfully amplified with PCR. The 5′ end of the RNA genome was amplified with the 5′ RACE system for rapid amplification of cDNA ends (Invitrogen, Carlsbad, CA, USA). The 3′ terminal nucleotide sequence of the *P. asiatica* L.–infecting virus was determined with 3′ RACE using the oligo(dT) primer 5′-GTTTTCCCAGTCACGAC(T)_15_-3′ (4, 5). All PCR fragments were cloned into the RBC T&A cloning vector (RBC Bioscience, Taipei, Taiwan) for sequencing by GenoTech Corp. (Daejeon, South Korea). The sequences were assembled with DNAMAN version 5.2.10 (Lynnon Biosoft, Quebec, Canada).

The complete nucleotide sequence of the HaMV isolate PA1 genome contains 9,499 nucleotides, excluding the poly(A) tail (GenBank accession no. KR091311). The potyviral genome is expressed as a polyprotein, which is cleaved by viral-encoded proteases into 10 functional proteins (6). Comparison of the complete nucleotide and polyprotein amino acid sequences of the HaMV PA1 and HaMV Ha-1 genomes showed 81.11% and 90.11% identities, respectively. Pairwise comparisons based on amino acid sequence alignments of 10 functional proteins between HaMV PA1 and 29 other potyviruses showed that the highest amino acid sequence identities with HaMV Ha-1 were 82.44% for P1, 91.68% for HC-Pro, 87.36% for P3, 90.57% for 6K1, 92.85% for CI, 88.68% for 6K2, 87.30% for VPg, 92.56% for NIa, 90.19% for NIb, and 91.64% for CP. Although HaMV PA1 is closely related to HaMV Ha-1 in terms of their sequence identities, the two viruses were isolated from different host plants and geographic regions.

### Accession number(s).

The complete genome sequence of *Habenaria mosaic virus* isolate PA1 has been deposited in GenBank under the accession number KR091311.

## References

[1] Huang DF, Xie MY, Yin JY, Nie SP, Tang YF, Xie XM, Zhou C 2009 Immunomodulatory activity of the seeds of *Plantago asiatica* L. J Ethnopharmacol 124:493–498. doi:10.1016/j.jep.2009.05.017. 19467312

[2] Kondo H, Maeda T, Gara IW, Chiba S, Maruyama K, Tamada T, Suzuki N 2014 Complete genome sequence of Habenaria mosaic virus, a new potyvirus infecting a terrestrial orchid (*Habenaria radiata*) in Japan. Arch Virol 159:163–166. doi:10.1007/s00705-013-1784-6. 23857506

[3] Gubler U, Hoffman BJ 1983 A simple and very efficient method for generating cDNA libraries. Gene 25:263–269. doi:10.1016/0378-1119(83)90230-5. 6198242

[4] Chen J, Chen J, Adams MJ 2001 A universal PCR primer to detect members of the *Potyviridae* and its use to examine the taxonomic status of several members of the family. Arch Virol 146:757–766. doi:10.1007/s007050170144. 11402861

[5] Zhao F, Lim S, Yoo RH, Lim HS, Kwon SY, Lee SH, Moon JS 2013 Complete genome sequence of a South Korean isolate of Brugmansia mosaic virus. Arch Virol 158:2019–2022. doi:10.1007/s00705-013-1693-8. 23584420

[6] Adams MJ, Antoniw JF, Fauquet CM 2005 Molecular criteria for genus and species discrimination within the family *Potyviridae*. Arch Virol 150:459–479. doi:10.1007/s00705-004-0440-6. 15592889

